# Do Concurrent Multiple Infections with High-Risk HPVs Carry a More Malignant Potential than a Single Infection in the Uterine Cervix?

**DOI:** 10.3390/jcm12196155

**Published:** 2023-09-24

**Authors:** Juhun Lee, Hyun Jung Lee

**Affiliations:** Department of Obstetrics and Gynecology, School of Medicine, Kyungpook National University, Kyungpook National University Hospital, Daegu 41944, Republic of Korea; gyjhlee@knu.ac.kr

**Keywords:** human papillomavirus, concurrent multiple HPV infections, cervical premalignancy, malignant potential, risk of carcinogenesis, cervical cancer

## Abstract

The high-risk human papillomavirus (HR-HPV) has been known as the most important carcinogen in uterine cervical carcinoma. However, there is limited evidence of the malignant potential of these concurrent multiple infections. This study included women who had undergone cervical conization. They underwent an HPV test by cervical swab within 12 months before the surgery. They were divided into two groups: one with a single infection with HR-HPV16 and the other with concurrent multiple infections with HR-HPVs, including genotype 16. Pathologic examination classified cases as CIS+ to assess and compare the malignant potential in both groups, including carcinoma in situ (CIS) and invasive carcinoma. Of the 220 patients infected with HR-HPV16, the single infection group consisted of 120 patients (54.5%), whereas the concurrent multiple infections consisted of 100 (45.5%) patients. The rates of HSIL were significantly higher in the concurrent multiple infection group. However, the odds ratio for CIS+ did not show a significant difference between both groups (1.417, 95% CI = 0.831–2.414, *p* = 0.200). The malignant potential was not significantly different between concurrent multiple infections with HR-HPVs, including 16, and a single infection with 16 in Korean women.

## 1. Introduction

Cervical cancer is the fourth most prevalent cancer among women worldwide and the fifth among Korean women [1,2]. Several studies have proved that persistent infection with high-risk human papillomavirus (HR-HPV) is the leading cause of cervical cancer [3,4,5,6,7]. Over 100 genotypes have been identified in HPV, of which approximately 20 are at a high risk of carcinogenesis [8]. Among these genotypes, 16 was found to be the most prevalent in many regions or countries, whereas other genotypes have different distributions [9,10,11,12,13,14]. Globally, HPV16 and HPV18 are the two most prevalent HR-HPVs in approximately 70% of invasive cervical cancers [10,14,15].

Since the approval of the quadrivalent HPV vaccine and bivalent HPV vaccine by the US Food and Drug Administration in 2006 and 2009, respectively, the efficacy of these vaccines has been demonstrated through many clinical trials [16]. The Korean government has included these HPV vaccinations in the national program since 2016 and provided support for bivalent or quadrivalent HPV vaccines for girls aged 12–17, which has resulted in an approximate vaccination rate of 70% among girls in the Republic of Korea [17].

HPV infects the basal layer of the squamous epithelium. Although most HPV infections (up to 90%) can be cleared by the immune system in the human body, approximately 10% become persistent [18]. However, based on a previous study, the rate of persistent HPV infection can vary with age, potentially reaching up to 30.6% in women aged 21–30 years [15]. Of the various products of several oncogenes of HPV, E6 and E7 play an important role in inducing malignant progression [19,20]. The oncoprotein E6 binds with p53 and inhibits the function of the tumor suppressor protein via ubiquitin-dependent degradation, whereas E7 activates cell proliferation via pRB inhibition [20,21,22].

According to recent studies in Korea, a single HR-HPV infection is more prevalent in premalignant lesions and invasive carcinoma of the uterine cervix than multiple HR-HPV infections [10,11]. Furthermore, previous studies reported that concurrent multiple infections with HPVs account for 20–40% [10,11,23,24]. It was also found that multiple infections frequently occur in young women due to various factors, including sexual activity [23,25,26]. However, this evidence is insufficient to demonstrate the malignant potential of concurrent multiple infections. The multiple infection groups in those studies seem to be very heterogeneous, or the researchers did not thoroughly evaluate the clinical significance.

In this study, we determined whether concurrent multiple infections have a higher risk of carcinogenesis than a single infection in the uterine cervix in Korean women.

## 2. Materials and Methods

### 2.1. Patients

**Figure 1:**
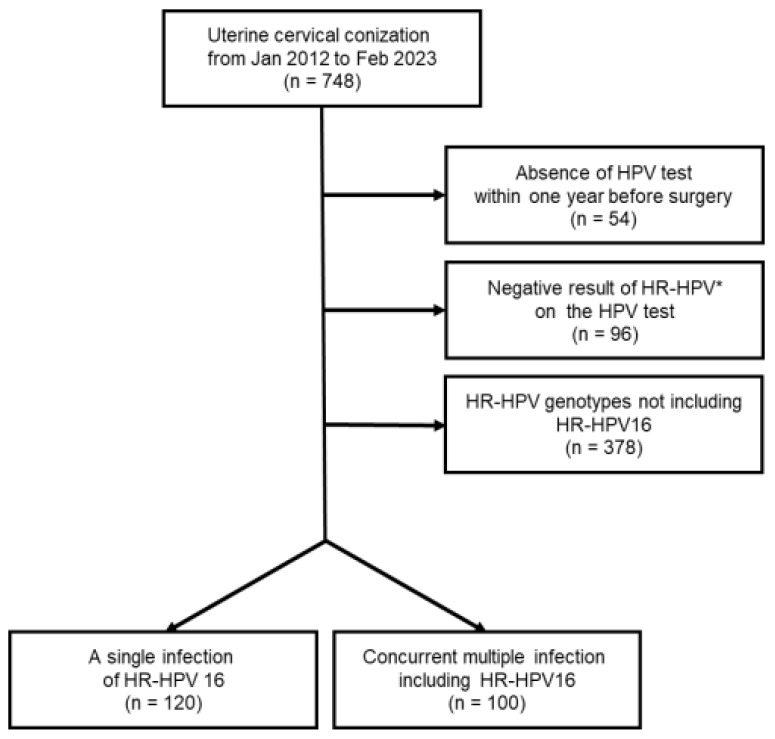
Flow diagram for patient selection (* HR-HPV: high-risk human papillomavirus).

We retrospectively reviewed 748 patients who underwent uterine cervical conization for the diagnosis or treatment of cervical premalignant lesions or carcinoma from January 2012 to February 2023 in Kyungpook National University Hospital (KNUH). A total of 55 patients were excluded due to the absence of an HPV test within 12 months before the surgery, whereas 96 patients were excluded due to the negative result on the HPV test or the absence of HR-HPVs. HR-HPV16 was most common among the remaining patients who were proven to be infected. Consequently, we excluded 378 patients who were not found to have HR-HPV16, because there was insufficient evidence to determine whether every HR-HPV genotype has the same malignant potential. Finally, we enrolled 220 patients in this study. The flow diagram for patient selection is presented in Figure 1. This study was approved by the institutional review board (KNUH 2023-06-015).

### 2.2. Initial and Final Diagnosis

The initial diagnosis was based on the pathologic result of cervical swab cytology along with the 2001 Bethesda System or cervical punch biopsy [27]. Acute or chronic cervicitis and reactive changes were considered normal. The atypical cells included atypical squamous cells (ASCs); ASCs of undetermined significance (ASCUS); ASCs, cannot exclude a high-grade lesion (ASC-H); atypical glandular cells (AGCs); and AGCs of undetermined significance (AGUS). On the other hand, the final diagnosis was based on the pathologic report of cervical conization. Acute or chronic inflammation was also considered normal. When the lesions of two different grades, for example, low-grade squamous intraepithelial lesion (LSIL) and high-grade squamous intraepithelial lesion (HSIL), were simultaneously detected, a diagnosis of higher malignant potential was made. The HSIL in the initial or final diagnosis did not include carcinoma in situ (CIS). CIS in the initial or final diagnosis also included some cases of cervical intraepithelial neoplasia 3, which had been diagnosed with CIS.

### 2.3. Classification of CIS+

To evaluate the malignant potential between both groups, one consisting of patients with a single HR-HPV16 infection and the other with concurrent multiple infections, including HR-HPV16, we categorized a subgroup of CIS+ based on the final diagnosis as described above. It encompassed CIS and invasive carcinoma, such as squamous cell carcinoma or endocervical adenocarcinoma.

### 2.4. Statistical Analysis

We compared and evaluated the variables between the single infection and concurrent multiple infection groups. The categorical variables between the groups were compared using the chi-squared test or Fisher’s exact test, whereas the continuous variables were compared using Student’s *t*-test. The ratio of HSIL+ or CIS+ between the groups was analyzed using logistic regression. A *p*-value of less than 0.05 was considered statistically significant. All statistical analyses were conducted using SPSS version 26 (IBM Corp., Armonk, NY, USA).

### 2.5. Uterine Cervical Conization

Loop electrosurgical excision was adopted for the uterine cervical conization. In this study, 15 surgeons performed the surgery. According to each surgeon’s decision, endocervical conization and/or endocervical curettage were also performed. Before surgery, colposcopy using acetic acid was employed to determine the resection margin.

### 2.6. HPV Test

The HPV test was performed using a uterine cervical swab. Both DNA microarray and real-time polymerase chain reaction (RT-PCR) were used to determine the status of HPV infection, as both methods were demonstrated to have similar validation [28,29]. The HPV tests were not centrally reviewed. Thus, the results in local medical institutions, not only in our institution, were also included in this study. In our institution, the HPV test has been performed and analyzed using PANA RealTyper^TM^ HPV kit (PANAGENE, Daejeon, Republic of Korea).

## 3. Results

**Figure 2:**
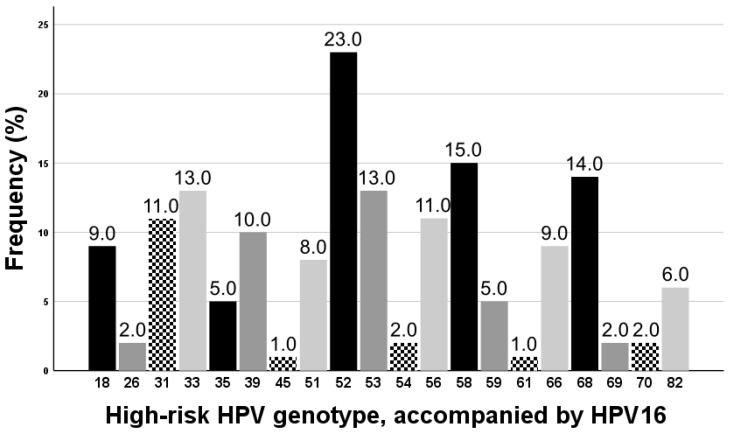
High-risk human papillomavirus (HR-HPV) genotype, accompanied by HR-HPV16, in concurrent multiple HR-HPV infection group. The frequency of HR-HPV16 was not shown in the graph because every patient had been found to have HR-HPV16. The frequency in the graph corresponds to the number detected in the HPV test, which was 100 in this group.

Of the included patients, 220 were found to be infected with HR-HPV16. Of these patients, 120 (54.5%) and 100 (45.5%) belonged to the single infection group and concurrent multiple infection group, respectively. Furthermore, the mean ages in both groups were 45.37 ± 12.81 years and 42.90 ± 15.84 years, respectively, indicating no significant difference (*p* = 0.212). In terms of the number of gravida, parity, and abortion, the single infection group showed significantly higher parity (1.63 ± 1.11 vs. 1.19 ± 1.32, *p* = 0.008), while there were no significant differences in terms of the number of gravida or abortion (*p* = 0.292 and *p* = 0.436, respectively). In the concurrent multiple infection group, the mean number of HR-HPVs infected concurrently was 2.63 ± 0.98 (range, 2–6); 62 (62.0%) patients had two genotypes, 22 (22.0%) had three, 9 (9.0%) had four, 5 (5.0%) had five, and 2 (2.0%) had six different HR-HPVs. When comparing the initial diagnoses, the concurrent multiple infection group exhibited significantly more HSIL than the single infection group (61 (61.0%) vs. 52 (43.3%), *p* = 0.010), whereas there were no significant differences between both groups (Table 1). Within the 100 cases of the concurrent multiple infection group, the five most commonly accompanied HR-HPV genotypes with HPV16, in descending order, were 52, 58, 68, 33, and 53 (23 (23.0%), 15 (15.0%), 14 (14.0%), 13 (13.0%), 13 (13.0%), respectively). The remaining information is shown in Figure 2.

Final diagnoses, determined through pathological examination of cervical conization, between the single infection group with HR-HPV16 and the concurrent multiple infections group with HR-HPVs, including HPV16, were also compared. The single infection group exhibited a significantly higher rate of normal cases (19 [15.8%] vs. 5 [5.0%], *p* = 0.015), whereas the concurrent multiple infection group showed a significantly higher rate of HSIL cases (33 [33.0%] vs. 19 [15.8%], *p* = 0.004). The presence of atypical cells, LSIL, CIS, or invasive carcinoma did not significantly differ between both groups (*p* = 0.628, *p* = 0.392, *p* = 0.499, and *p* = 0.352, respectively). The single infection group showed an insignificantly higher rate of CIS+ in comparison to the concurrent multiple infection group (68 [56.7%] vs. 48 [48.0%], *p* = 0.223) (Table 2).

In the logistic regression used to compare the malignant potential between the groups, no significant difference was observed. In the final diagnosis, the odds ratio (OR) for CIS+ was 1.417 (95% confidence interval [CI] = 0.831–2.414, *p* = 0.200).

## 4. Discussion

Among the patients included in this study, those with concurrent multiple infections, including HR-HPV16, did not significantly influence the malignant potential compared with the single infection with HR-HPV16 in young and old women when reviewed comprehensively.

The result of our study can be used by physicians to explain the malignant potential of concurrent multiple infections with HR-HPVs to their patients. Concurrent multiple infections, including HPV16, did not have a significantly increased risk of carcinogenesis in the uterine cervix compared with a single infection with HR-HPV16. However, physicians need to monitor the patients showing concurrent multiple HR-HPV infections, as they can have a higher prevalence of HSIL in the uterine cervix, especially in middle-aged or old women.

We could not find any previous study to evaluate the malignant potential of concurrent multiple infections with HR-HPVs. Some recent studies in the Republic of Korea have demonstrated a significantly lower prevalence of concurrent multiple infections than a single infection with HR-HPV [10,11]. However, these studies did not compare the malignant potential between the groups.

Subgroup analysis was performed to control the influence of age on carcinogenesis. The cohort was divided into two groups, one consisting of patients aged ≤40 years (*n* = 95) and the other comprising those >40 (*n* = 125). We compared the rates of final diagnoses and CIS+ between the single infection and concurrent multiple infection groups.

In the subgroup of ≤40 years, the results of all final diagnoses exhibited no significant differences between the two groups. The rate of CIS+ was also similar (27 (60.0%) vs. 25 (25.0%), *p* = 0.410). The OR for CIS+ was not significant between groups (OR = 1.417, 95% CI = 0.831–2.414, *p* = 0.200).

In the subgroup of >40 years, HSIL was significantly more prevalent in the concurrent multiple infection group (13 (26.0%) vs. 8 (10.7%), *p* = 0.030), which differed from other final diagnoses. However, the rate of CIS+ was not significantly different between the single infection group and multiple infection group (41 (54.7%) vs. 23 (46.0%), *p* = 0.366). The OR for CIS+ was not significant between both groups, either (OR = 1.416, 95% CI = 0.690–2.903, *p* = 0.343, respectively).

In this subgroup analysis, we also compared the prevalence of concurrent multiple HR-HPV infections between the subgroup of ≤40 years and >40 years. Among those aged ≤ 40 years, 50 (52.6%) had concurrent multiple HR-HPV infection, including genotype 16, whereas 50 (40.0%) were found among those aged > 40 years. The difference was not statistically significant in the Chi-square test (*p* = 0.076), and the OR was 0.600 (95% CI = 0.350–1.028). Young women showed a tendency to have more concurrent multiple infections, which was similar to the findings in some previous studies [23,25,26]. However, as described above, this tendency does not seem to result in a significantly higher malignant potential in young women.

The strength of this study was the homogeneous cohort achieved through a thorough exclusion, which enabled us to compare the malignant potential of HR-HPV between the single infection and concurrent multiple infection groups with less bias compared with previous studies.

This study also has some limitations. First, this was a retrospective study involving a small sample size from a single center. Second, in the cohort, we could not demonstrate persistent infection with HR-HPVs, which is known as a critical factor for carcinogenesis in cervical cancer [7,30]. Third, we could not control the influence of the HPV vaccination as the vaccination history had not been reviewed. Nevertheless, it would be intriguing to investigate the correlation between HPV vaccination status and HPV infection status, such as concurrent multiple infection, as observed in this study. Finally, although the two methods for HPV test, namely, DNA microarray and RT-PCR, used in this study demonstrated similar validation, heterogeneity may arise. Furthermore, the HPV test was not centrally reviewed.

## 5. Conclusions

Premalignant lesions in the uterine cervix can result significantly more frequently in Korean women aged > 40 years who exhibit concurrent multiple HR-HPV infections, including genotype 16, in contrast to single HR-HPV16 infection. However, the prevalence of invasive carcinoma or carcinoma in situ in the uterine cervix was not significantly different between the two groups, regardless of age. This result should be validated in a larger cohort.

## Figures and Tables

**Table 1:** Comparison of the characteristics and clinical factors between the group with single infection with HR-HPV16 and the group with concurrent multiple infections with HR-HPVs, including HPV16.

	Single Infection Group (*n* = 120)	Concurrent Multiple Infection Group (*n* = 100)	*p*-Value
Age (yrs)	45.37 ± 12.81	42.90 ± 15.84	0.212 *
Gravida (*n*)	2.58 ± 1.74	2.28 ± 2.39	0.292 *
Parity (*n*)	1.63 ± 1.11	1.19 ± 1.32	0.008 *
Abortion (*n*)	0.96 ± 0.99	1.09 ± 1.42	0.436 *
Number of infection with high-risk HPVs (range)	1	2.63 ± 0.98	<0.001 *
6 genotypes		2 (2.0%)	
5 genotypes		5 (5.0%)	
4 genotypes		9 (9.0%)	
3 genotypes		22 (22.0%)	
2 genotypes		62 (62.0%)	
Initial diagnosis ^‡^ (*n*)			
Normal	1 (0.8%)	1 (1.0%)	>0.999 ^†^
Atypical cells	9 (7.5%)	4 (4.0%)	0.391 ^†^
LSIL	10 (8.3%)	5 (5.0%)	0.424 ^†^
HSIL ^§^	52 (43.3%)	61 (61.0%)	0.010 ^†^
Carcinoma in situ ^¶^	43 (35.8%)	25 (25.0%)	0.107 ^†^
Invasive carcinoma	5 (4.2%)	4 (4.0%)	>0.999 ^†^

Data are expressed as *n* (%) and mean ± standard deviation. *: statistical significance was evaluated using Student’s *t*-test. ^†^: statistical significance was evaluated using chi-squared test. ^‡^: based on the pathologic result of cervical swab cytology or biopsy. ^§^: included CIN2 and CIN3, which is not the carcinoma in situ. ^¶^: also included some CIN3, which is diagnosed with carcinoma in situ. Abbreviations: LSIL: low-grade squamous intraepithelial lesion, HSIL: high-grade squamous intraepithelial lesion, and CIN: cervical intraepithelial neoplasm.

**Table 2:** Comparison of the final diagnoses based on the pathologic examinations between the group with single infection with HR-HPV16 and the group with concurrent multiple infections with HR-HPVs, including HPV16.

	Whole Group			Subgroup (≤40 Years)			Subgroup (>40 Years)		
	Single Infection (*n* = 120)	Multiple Infection (*n* = 100)	*p*-Value	Single Infection (*n* = 45)	Multiple Infection (*n* = 50)	*p*-Value	Single Infection (*n* = 75)	Multiple Infection (*n* = 50)	*p*-Value
Normal	19 (15.8%)	5 (5.0%)	0.015	5 (11.1%)	1 (2.0%)	0.098	14 (18.7%)	4 (8.0%)	0.122
Atypical cells	3 (2.5%)	1 (1.0%)	0.628	0 (0%)	1 (2.0%)	>0.999	3 (4.0%)	0 (0%)	0.274
LSIL	11 (9.2%)	13 (13.0%)	0.392	2 (4.4%)	3 (6.0%)	>0.999	9 (12.0%)	10 (20.0%)	0.309
HSIL ^†^	19 (15.8%)	33 (33.0%)	0.004 *	11 (24.4%)	20 (40.0%)	0.128	8 (10.7%)	13 (26.0%)	0.030 *
Carcinoma in situ ^‡^	60 (50.0%)	45 (45.0%)	0.499	25 (55.6%)	24 (48.0%)	0.539	35 (46.7%)	21 (42.0%)	0.714
Invasive carcinoma	8 (6.7%)	3 (3.0%)	0.352	2 (4.4%)	1 (2.0%)	0.602	6 (8.0%)	2 (4.0%)	0.474
HSIL+	87 (72.5%)	81 (81.0%)	0.154	38 (84.4%)	45 (90.0%)	0.540	49 (65.3%)	36 (72.0%)	0.557
CIS+	68 (56.7%)	48 (48.0%)	0.223	27 (60.0%)	25 (25.0%)	0.410	41 (54.7%)	23 (46.0%)	0.366

Data are expressed as *n* (%). Statistical significance was evaluated using chi-squared test. *: Statistically significant. ^†^: Included CIN2 and CIN3, which is not the carcinoma in situ. ^‡^: Included also some CIN3, which is diagnosed with the carcinoma in situ. Abbreviations: LSIL: low-grade squamous intraepithelial lesion, HSIL: high-grade squamous intraepithelial lesion, and CIN: cervical intraepithelial neoplasm.

## Data Availability

The data in this study are available upon a reasonable request to the corresponding author.
